# The urgent need for well-being programs in medical residencies in Brazil

**DOI:** 10.1590/1516-3180.2023.0274.R1.29012025

**Published:** 2025-05-19

**Authors:** Marcelo Bruno Generoso, Pedro Shiozawa, Ricardo Uchida, Marsal Sanches

**Affiliations:** I Mental Health Department, Faculdade de Ciências Médicas da Santa Casa de São Paulo, São Paulo (SP), Brazil.; II Mental Health Department, Faculdade de Ciências Médicas da Santa Casa de São Paulo, São Paulo (SP), Brazil.; III Mental Health Department, Faculdade de Ciências Médicas da Santa Casa de São Paulo, São Paulo (SP), Brazil.; IV Department of Psychiatry and Behavioral Sciences at McGovern Medical School-UT Health Science Center, Houston, Texas, United States.

Dear Editor,

Medical residency is a demanding phase characterized by an intense workload, high cognitive and emotional demands, and the need to assimilate a large volume of theoretical and practical knowledge. This period also coincides with major personal transitions, including increasing responsibilities and a growing sense of independence.

According to the latest medical demographic census, Brazil had 41,853 physicians enrolled in residency programs in 2021, accounting for 8% of all physicians in the country. Of these, 58% began their residency immediately after graduation or within 1 year, suggesting a limited time to reflect on career choices or explore specialized fields—potentially increasing the risk of unmet expectations or professional dissatisfaction.^1^

Additionally, approximately 70% of residents reported working 20 extra hours beyond their regular duties, while 30% had student loans. Furthermore, 68% were enrolled in programs located in cities or states different from where they earned their degrees. The survey included residents up to 35 years of age.

Taken together, these findings present Brazilian medical residents as recently graduated physicians with limited clinical experience entering the workforce under intense pressure. Many work 20 additional hours beyond the mandated 60-h weekly load, often away from their families and support networks, while managing financial obligations such as student loans.

These stressors are compounded by challenges inherent to residency, including heavy workloads, frequent rotation across departments, emotionally intense situations, and consistent exposure to trauma and human suffering. Residents also experience limited time with family and friends, high responsibility, and the emotional burden of dealing with the potential consequences of clinical errors.^2^

A recent meta-analysis involving 22,778 residents reported an overall burnout prevalence of 51.0% (95% CI: 45.0–57.0%, I^2^ = 97%), consistent with a Brazilian survey of 1,419 residents that found positive screenings for depression (46.9%), anxiety disorders (56.6%), and burnout (37%).^3,4^ Beyond the impact on individual well-being, this psychological distress may also compromise patient care. Currently, Brazil’s National Medical Residency Commission (CNRM) does not regulate strategies to promote resident well-being or prevent mental illness.

Effective burnout prevention strategies among physicians fall into two categories: structural interventions—such as adjusting rotation schedules, improving workflows, and reducing shift loads when possible—and individual-level approaches. The latter includes peer support, communication skills and stress management training, self-care promotion, and programs that foster a sense of community and belonging.^5^

To better understand this issue, it is important to consider the method of medical residency candidate selection in Brazil. Each teaching institution independently conducts this process through a public selection system that must comply with the CNRM’s guidelines and include at least one stage of assessing theoretical knowledge.^6^ Additional stages—such as clinical practice evaluation and curricular assessment—are optional and may contribute a maximum of 40% and 10%, respectively, to the final score. Moreover, candidate interviews are not included, and the curricular assessment stage consists solely of a documental checklist.

The optional nature of these stages, along with the absence of interviews and the inability to evaluate attributes beyond certifications, may hinder optimal alignment between candidates and residency programs. Interviews could help clarify program-specific details, address uncertainties, and align expectations.

The high prevalence of burnout and psychological distress among residents presents a significant and global concern. Nevertheless, effective strategies can be integrated into residency programs to help mitigate these outcomes. We urge Brazilian program coordinators to carefully evaluate the current landscape and implement context-specific measures to address this pressing challenge.
